# Antioxidative and Skin Protective Effects of *Canarium subulatum* Methanol Extract on Keratinocytes

**DOI:** 10.1155/2021/6692838

**Published:** 2021-03-10

**Authors:** So-Hyeon Hwang, Ji Hye Kim, Eunju Choi, Sang Hee Park, Jae Youl Cho

**Affiliations:** ^1^Department of Integrative Biotechnology and Biomedical Institute for Convergence at SKKU (BICS), Sungkyunkwan University, Suwon 16419, Republic of Korea; ^2^Department of Biocosmetics, Sungkyunkwan University, Suwon 16419, Republic of Korea

## Abstract

*Canarium subulatum* is a traditional medical herb used in South Asia. Recently, the anti-inflammatory effects of *C. subulatum* methanol extract (Cs-ME) have been reported; however, the effect of Cs-ME on skin physiology has not yet been elucidated. Therefore, in this study, we evaluated the protective effect of Cs-ME on UV-induced skin aging and cell death as well as the reinforcing effect on the skin barrier. According to viable cell counting and MTT assays, Cs-ME significantly reduced UV-evoked HaCaT cell death. Cs-ME blocked reactive oxygen species (ROS) generation in UV-irradiated HaCaT cells and showed radical scavenging activity against DPPH and ABTS. In addition, H_2_O_2_-induced cell death was inhibited by Cs-ME, indicating that Cs-ME protects cells from UV-derived cell death through the suppression of ROS. PCR analysis revealed that Cs-ME diminished the expression of aging-related HYAL-1 and MMP-1 genes in UV-treated HaCaT cells. Elevated HYAL-1 and MMP-1 mRNA expression in H_2_O_2_-stimulated HaCaT cells was also decreased by Cs-ME, suggesting that Cs-ME exerts antiaging activity via the inhibition of ROS. Expression of skin barrier components including filaggrin and hyaluronic acid synthase-1 was increased by Cs-ME and was modulated by ERK/p38-AP-1 signaling. Collectively, our data show that Cs-ME has cytoprotective and antiaging activity based on antioxidant properties. Furthermore, Cs-ME exerts skin barrier protective ability by regulating the AP-1 signaling pathway. Therefore, Cs-ME has the potential for use as an ingredient in cosmetics to protect the skin from UV irradiation, prevent photoaging, and strengthen the skin barrier.

## 1. Introduction

The epidermis serves as a barrier to protect the body from external irritation, pathogen invasion, and water loss [1]. Approximately 90% of epidermal cells are keratinocytes, which form the skin barrier by producing structural proteins such as filaggrin (FLG) and transglutaminase-1 (TGM-1) as well as proinflammatory mediators [2–4]. Keratinocytes also play a role in maintaining skin hydration via producing hyaluronic acid synthase (HAS), which biosynthesizes hyaluronic acid (HA) that is one of several biomolecules related to skin hydration [5]. There are three types of HASs (−1, −2, and −3), which synthesize diverse lengths of HA through different enzyme activities [6].

Mitogen-activated protein kinases (MAPKs) control various cellular responses including proliferation, differentiation, cell survival, apoptosis, mitosis, and gene expression [7]. Four mammalian MAPK components including extracellular signal-regulated kinases 1 and 2 (ERK1/2), c-Jun N-terminal kinase (JNK), p38, and ERK5 have been identified [8]. Among the MAPKs, EKK1/2, JNK, and p38, which were relatively well understood, contribute to activation of the activator protein 1 (AP-1) transcription factor in response to various extracellular stimuli [9]. In particular, the MAPK-AP-1 signaling pathway is known as a key player in the regulation of proinflammatory cytokine expression in a variety of cells, including keratinocytes [10]. In addition, studies have reported that MAPKs are also associated with FLG and HAS expression in keratinocytes [11, 12].

Skin aging is mainly caused by two independent processes: photoaging or intrinsic (age-dependent) aging [13]. Photoaging refers to skin aging due to excessive ultraviolet (UV) irradiation exposure, which is caused by increased reactive oxygen species (ROS) production [14]. UV regulates the development of ROS through a variety of mechanisms such as increasing nitric oxide synthase (NOS) synthesis, decreasing protein kinase C (PKC) expression, and regulating enzyme catalase activity [15, 16]. When UV produces a large amount of ROS that antioxidant mechanisms cannot remove, oxidative stress is induced resulting in cell death through cellular damage and apoptosis [17]. In addition, UV-induced ROS reduces skin elasticity by increasing the expression of hyaluronidases (HYALs) and matrix metalloproteinases (MMPs), which are responsible for collagen, elastin, and HA degradation, the main components of the extracellular matrix [18]. Therefore, reagents that can inhibit the production of or quickly remove ROS can be used as antiaging ingredients to prevent photoaging.

The genus *Canarium* is comprised of about 78 species of tropical and subtropical trees and *Canarium subulatum* is a tropical tree species belonging to the family Burseraceae [19]. The fruit of *C. subulatum* Guillaumin has been used as an expectorant, and white aromatic latex flowing from bark wounds has been utilized to treat pruritus [20]. Although the anti-inflammation and antiherpetic activities of *C. subulatum* Guillaumin extract have been reported, there are no reports on skin bioactivity in human keratinocytes [20, 21]. Therefore, we evaluated the effect of *C. subulatum* Guillaumin methanol extract (Cs-ME) on UV-induced skin aging and cell damage as well as skin protection.

## 2. Materials and Methods

### 2.1. Materials

Cs-ME with flavonoids including quercetin (0.115%), luteolin (0.088%), and kaempferol (0.031%) as active components [21] was obtained from the Plant Extract Bank of the Plant Diversity Research Centre (Daejeon, Korea). HaCaT and HEK293 T cells were purchased from ATCC (Rockville, MD, USA). Dulbecco's modified Eagle's medium (DMEM), fetal bovine serum (FBS), phosphate-buffered saline (PBS), penicillin-streptomycin, bovine serum albumin (BSA), 1-diphenyl-2-picryl-dydrazyl (DPPH), 2,2'-azino-bis(3-ethylbenzothiazoline-6-sulphonic acid) diammonium salt (ABTS), potassium sulfate, and ascorbic acid were purchased from Hyclone (Grand Island, NY, USA). 3-(4,5-Dimethylthiazol-2-yl)-2,5-diphenyl tetrazolium bromide (MTT) and U0126 were purchased from Sigma Aldrich Chemical Co. (St. Louis, MO, USA). The MTT stopping solution was prepared by adding 10% sodium dodecyl sulfate (SDS) to hydrochloric acid (HCl). The TRI reagent was purchased from Molecular Research Center Inc. (Cincinnati, OH, USA). MuLV reverse transcriptase (RT) and H2-DCFDA stain were purchased from Thermo Fisher Scientific (Waltham, MA, USA). Semiquantitative reverse transcription-polymerase chain reaction (RT-PCR) forward and reverse primers for each target were purchased from Bioneer Inc. (Daejeon, Korea). The PCR premix was purchased from PCR Biosystems Ltd. (London, United Kingdom). FBS, PBS, and TRIzol reagents were purchased from GIBCO (Grand Island, NY, USA). Bradford solution, polyvinylidene fluoride (PVDF) membranes, and enhanced chemiluminescence (ECL) were purchased from Bio-Rad (Hercules, CA, USA). Antibodies against total or phosphorylated forms of extracellular signal-regulated kinase (ERK), JNK, p38, and *β*-actin were purchased from Cell Signaling Technology (Beverly, MA, USA) and Santa Cruz Biotechnology (Santa Cruz, CA, USA).

### 2.2. Cell Culture

HaCaT cells were cultured in DMEM with 10% FBS and 1% penicillin-streptomycin at 37°C. HEK293 T cells were cultured in DMEM with 5% FBS and 1% penicillin-streptomycin. Both cell lines were incubated in a 5% CO_2_ humidified incubator.

### 2.3. Treatment of Cm-ME

For in vitro experiment, Cs-ME was dissolved in 100% dimethyl sulfoxide (DMSO) to make stock solutions (12.5, 25, 50, 100, and 200 mg/mL). To prepare working solutions (12.5, 25, 50, 100, and 200 *μ*g/mL) of Cs-ME, DMEM with 5% FBS was used. Normal and control groups (UV, DPPH, ABTS, or H_2_O_2_ alone) were treated with the same amount of MDSO (0.1%).

### 2.4. DPPH Assay

The 1,1-diphenyl-2-picrylhydrazyl (DPPH) assays were performed to measure antioxidant activity in vitro [22]. A total of 250 *μ*M DPPH was prepared in 96-well plates and 50 and 100 *μ*g/mL of Cs-ME were added. Ascorbic acid (100 *μ*M) was used as the positive control. Absorbance at 517 nm was measured with a spectrophotometer (Spectramax 250 microplate reader, Marshall Scientific, USA). The DPPH radical scavenging effect was calculated as follows:

(1) $$\begin{array}{c} \text{DPPH scavenging effect}\left(\%\right)=\frac{A_{0}-A_{1}}{A_{0}}\times 100\left(\%\right), \end{array}$$

where *A*_0_ is the absorbance of DPPH and *A*_1_ is the absorbance of samples.

### 2.5. ABTS Assay

The ABTS radical scavenging assay was also performed to measure antioxidant activity in vitro [22]. A 1 : 1(v) ratio mixture of 2.4 mM potassium persulfate and 7 mM ABTS was prepared, and the solution was incubated at room temperature for 24 h to generate ABTS radicals. After the solution darkened, it was diluted with PBS and transferred to the wells of a 96-well plate. Cs-ME (50 and 100 *μ*g/mL) was added to each well, and ascorbic acid (100 *μ*M) was used as the positive control. Absorbance at 710 nm was measured with a spectrophotometer (Spectramax 250 microplate reader, Marshall Scientific, USA). The ABTS radical scavenging effect was calculated as follows:

(2) $$\begin{array}{c} \text{ABTS scavenging effect}\left(\%\right)=\frac{A_{0}-A_{1}}{A_{0}}\times 100\left(\%\right), \end{array}$$

where *A*_0_ is the absorbance of ABTS and *A*_1_ is the absorbance of samples.

### 2.6. Cell Viability Assay

HaCaT cells were plated at 5 × 10^4^ cells/well in 96-well plates. After 24 h, 50, 100, or 200 *μ*g/mL Cs-ME was added to respective wells and incubated for 24 h. A 100 *μ*L volume of media was removed from each well. Subsequently, 10 *μ*L MTT solution was added to each well and incubated for 4 h as previously reported [23]. Then, 100 *μ*L MTT stopping solution was added and the absorbance at 570 nm was measured using a spectrophotometer (Spectramax 250 microplate reader, Marshall Scientific, USA).

### 2.7. UVB Irradiation

HaCaT cells were seeded in 6-well plates at a density of 7 × 10^5^ cells/well followed by irradiation with 30 or 50 mJ/cm^2^ UVB using a BLX-312 Bio-Link crosslinker (Vilber Lourmat, Collegien, France) lamp as reported previously [24]. Consequently, cells were treated with Cs-ME and incubated for 24 h.

### 2.8. Viable Cell Counting Assay

HaCaT cells were seeded at a density of 4 × 10^5^ cells/mL in 6-well plates and treated with Cs-ME (0–100 *μ*g/mL) after UVB irradiation. After 24 h, cell images were captured using an inverted phase-contrast microscope (Olympus Co., Tokyo, Japan) with a video camera equipped with National Institutes of Health (NIH) imaging software. Three images from different areas were captured, and viable cells that adhered to the well were counted.

### 2.9. H_2_O_2_ Treatment

HaCaT cells were seeded in 6-well plates at a density of 7 × 10^5^ cells/well. A total of 50 *μ*M H_2_O_2_ was added to each plate, and cells were incubated for 24 h.

### 2.10. ROS Generation Assay

The 2',7'-dihydro-dichlorofluorescein diacetate (H2-DCFDA) assay was used to evaluate levels of ROS inside cells [25]. HaCaT cells were cultured at a density of 4 × 10^5^ cells/well in 12-well plates and irradiated with UV (30 mJ/cm^2^). Cells were incubated with Cs-ME (0, 50, 100 *μ*g/mL) or retinol (10 *μ*g/mL) for 24 h. Cells were washed with cold PBS to slow metabolism and were stained with 50 *μ*M DCF stain for 30 min without exposure to light. Cells were fixed for 20 min and analyzed using a Nikon Eclipse Ti (Nikon, Japan) fluorescence microscope. Mean fluorescence intensity (MFI) values were measured and the ROS generating cells were counted.

### 2.11. RT-PCR Analysis

HYAL-1, HYAL-2, HYAL-3, MMP-1, MMP-3, MMP-9, FLG, TGM-1, HAS-1, HAS-2, HAS-3, and GAPDH mRNA expression levels were determined quantitatively by RT-PCR. Total RNA was isolated with TRI reagent according to the manufacturer's instructions. cDNA was synthesized from 1 *μ*g total RNA using MuLV RT according to the manufacturer's instructions [26, 27]. The sequences of primers are listed in Table 1.

### 2.12. Immunoblotting Assay

Immunoblotting was performed to measure levels of phosphorylated and total forms of ERK, JNK, p38, and *β*-actin [28]. HaCaT cells were treated with Cs-ME (0, 50, and 100 *μ*g/mL) or retinol (10 *μ*g/mL) as the positive control for 24 h. Cells were lysed with lysis buffer, and cell debris was removed by centrifugation. Protein samples were separated by sodium dodecyl sulfate-polyacrylamide gel electrophoresis (SDS-PAGE) and were transferred to polyvinylidene fluoride membranes. The membranes were incubated with primary and secondary antibodies and detection was performed using enhanced chemiluminescence (ECL). The MFI values were measured.

### 2.13. Luciferase Reporter Gene Assay

HEK293 T cells (5 × 10^4^ cells/mL) were transfected with AP-1-Luc plasmids and *β*-galactosidase plasmids for 24 h using PEI as a transfection reagent. After 24 h, the cells were treated with either Cs-ME (0–100 *μ*g/mL) or retinol (10 *μ*g/mL) as the positive control for another 24 h. Next, cells were harvested and lysed by freezing and thawing. Then luciferase lysis buffer was added. Luciferase activity was measured using the Luciferase Assay System.

### 2.14. Statistical Analysis

All data presented in this study are expressed as the mean ± standard deviation (SD) from 3, 4, or 6 independent experiments as indicated. Statistical analyses were performed using the Kruskal-Wallis and Mann-Whitney tests. All statistical analyses were conducted with SPSS software (SPSS Inc., Chicago, IL, USA) and *p* values below 0.05 were considered statistically significant.

## 3. Results

### 3.1. Cytoprotective Effects of Cs-ME on UV-Exposed HaCaT Cells

**Figure 1:**
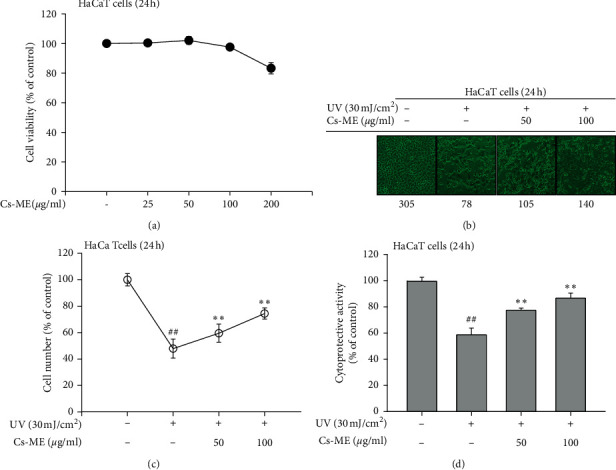
Protective effects of Cs-ME on cell death in UV-irradiated HaCaT cells. (a) Cytotoxicity of Cs-ME was assessed by MTT assays in HaCaT cells. (b and c) Cytoprotective effect of Cs-ME was evaluated by observing viable cell number via microscopy in UV-irradiated HaCaT cells. Three images from different regions in each group are pictured. Representative images are presented in (b). Viable cells that adhered to the well were counted (c). (d) The protective ability of Cs-ME against UV-induced cell death was evaluated by MTT assay in HaCaT cells. ^##^*p* < 0.01 compared to the normal group; ^*∗*^*p* < 0.05 and ^*∗∗*^*p* < 0.01 compared to the control group.

Since 100 *μ*g/mL of Cs-ME showed almost maximum NO inhibitory activity in LPS-treated RAW264.7 cells [21], we chose several doses from 12.5 to 100 *μ*g/mL of Cs-ME in this study. The cytotoxicity of Cs-ME was confirmed before efficacy evaluation. As shown in Figure 1(a), no cytotoxic effects were identified up to 100 *μ*g/ml Cs-ME. Subsequently, UV-irradiated HaCaT cells were treated with Cs-ME to study the protective effects of Cs-ME. Microscopic observation showed a significant decrease in the number of UV-treated HaCaT cells, whereas the number of cells in the Cs-ME treatment group increased (Figures 1(b) and 1(c)). MTT assays were performed to confirm the effect of Cs-ME on cell viability under UV-irradiated conditions. As a result, it was observed that Cs-ME reduced UV irradiation-induced cell death (Figure 1(d)), suggesting that Cs-ME has cytoprotective ability under UV exposure conditions.

### 3.2. Antioxidant Effects of Cs-ME

**Figure 2:**
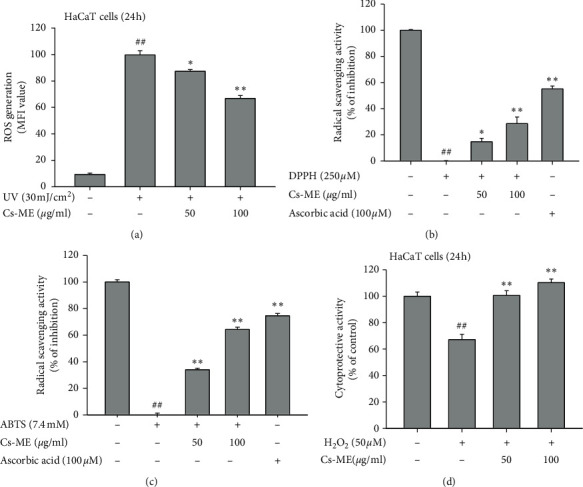
Antioxidant property of Cs-ME. (a) ROS generation in HaCaT cells treated with UVB and Cs-ME was analyzed via the H2-DCFDA staining method. H2-DCFDA intensity signal was quantified using ImageJ. (B and C) The radical scavenging activity of Cs-ME was measured by DPPH assay (b) and ABTS assay (c) in HaCaT cells. Ascorbic acid was used as the positive control. (d) Protective effect of Cs-ME on ROS-induced cell death was examined by MTT assay in HaCaT cells pretreated with H_2_O_2_ for 24 h. ^##^*p* < 0.01 compared to the normal group; ^*∗*^*p* < 0.05 and ^*∗∗*^*p* < 0.01 compared to the control group.

Cells generate ROS through diverse mechanisms when exposed to UV, and ROS have been reported to induce cell death [29]. Therefore, we examined the effect of Cs-ME on ROS generation. ROS production by UV was reduced with Cs-ME treatment in a dose-dependent manner (Figure 2(a) ). The antioxidant activity was also evaluated in a cell-free system. In the 1,1-diphenyl-picrylhydrazyl (DPPH) assay, Cs-ME showed 14.8% and 28.7% radical scavenging activity at 50 *μ*g/ml and 100 *μ*g/ml treatments, respectively (Figure 2(b)). ABTS radicals were removed by 34.1% and 64.4% at 50 *μ*g/ml and 100 *μ*g/ml Cs-ME, respectively (Figure 2(c)). These results suggest that although Cs-ME has a lower radical scavenging effect than the ascorbic acid control, it has significant antioxidant effects considering that it is a natural product. To determine whether the cytoprotective effect of Cs-ME on UV irradiation observed in Figure 1 was due to the regulation of ROS, the inhibitory effect of Cs-ME was evaluated in H_2_O_2_-treated HaCaT cells. Cell death induced by H_2_O_2_ was completely blocked by Cs-ME treatment, indicating that the Cs-ME exhibits cell-protective ability through ROS regulation (Figure 2(d)).

### 3.3. Antiaging Effect of Cs-ME on UV-Treated HaCaT Cells

**Figure 3:**
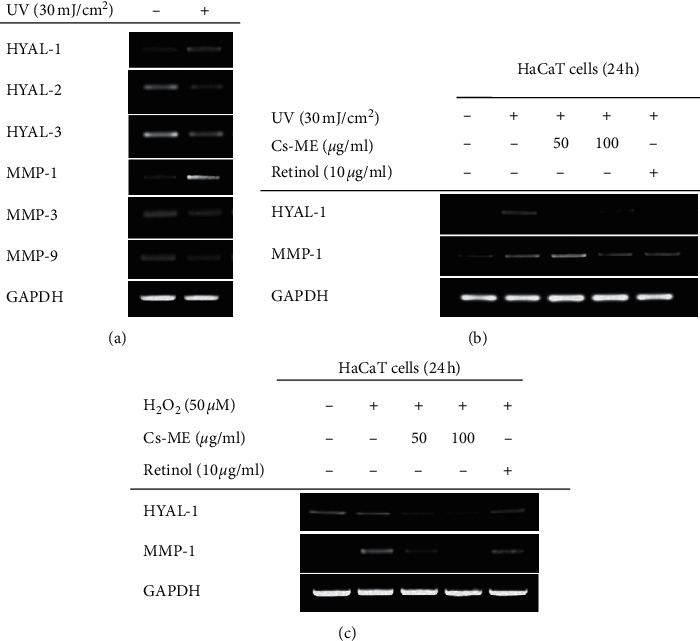
Antiaging effect of Cs-ME on UV-treated HaCaT cells. (a) PCR analysis was performed to identify HYAL and MMP genes that were overexpressed under UV irradiation conditions. (b) The inhibitory effect of Cs-ME on HYAL-1 and MMP-1 gene expression was validated by PCR analysis. Retinol, a powerful ingredient for antiaging in skin, was utilized as a positive control. (c) The effect of Cs-ME on ROS-induced HYAL-1 and MMP-1 expression was studied in H_2_O_2_-treated HaCaT cells through PCR analysis. Retinol was used as the positive control.

Emerging evidence indicates that UV-induced ROS is closely related to skin aging [30–32]. Therefore, we examined the effect of Cs-ME on UV-induced skin aging by observing alterations in HYAL and MMP gene expression. In our study, only HAYL-1 and MMP-1 were induced by UV irradiation in HaCaT cells (Figure 3(a) ). Cs-ME decreased the expression of UV-induced HAYL-1 and MMP-1 levels (Figure 3(b)). In addition, H_2_O_2_-induced HYAL-1 and MMP-1 gene expression was also reduced by Cs-ME (Figure 3(c)). These results indicate that Cs-ME has antiaging ability and this efficacy is derived from ROS inhibition.

### 3.4. The Effect of Cs-ME on Skin Barrier Function

**Figure 4:**
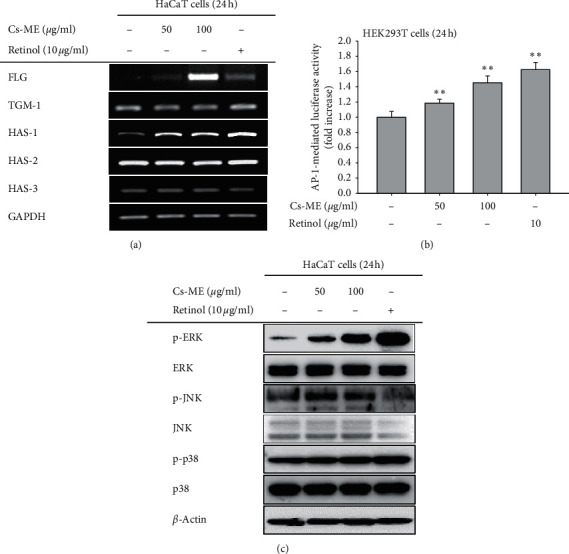
Protective effect of Cs-ME on the skin barrier. (a) To evaluate the skin barrier enhancement effect of Cs-ME, mRNA expression levels of FLG, TGM-1, and HAS epidermal components were analyzed by PCR in Cs-ME-treated HaCaT cells. Retinol treatment was used as the positive control. (b) The promoter activity of transcription factor AP-1 was determined by a reporter gene assay. HEK293 cells were transfected with AP-1-Luc (1 *μ*g/mL) and *β*-gal plasmids in the presence or absence of Cs-ME (50 or 100 *μ*g/mL) for 24 h. (c) The protein levels of phospho- and total forms of ERK, JNK, p38, and *β*-actin in whole-cell lysates of Cs-ME-treated HaCaT cells were measured by immunoblotting analysis. ^##^*p* < 0.01 compared to the normal group; ^*∗*^*p* < 0.05 compared to the control group.

The expression levels of FLG, TGM-1, and HAS genes were observed to evaluate the influence of Cs-ME on skin barrier function. Among the targets, expression of FLG and HAS-1, but not TGM-1, HAS-2, and HAS-3, was increased in Cs-ME (50 and 100 *μ*g/ml)-treated HaCaT cells (Figure 4(a) ). As it has been reported that the expression of FLG and HAS-1 genes is regulated by the transcription factor AP-1 [33], we tested if Cs-ME alters AP-1 activity. Cs-ME dose-dependently upregulated AP-1 luciferase activity (Figure 4(b)). The effect of Cs-ME on MAPKs (ERK, JNK, and p38), which are AP-1 activators, was also assessed. Cs-ME elevated phosphorylation of ERK and p38 but not JNK (Figure 4(c)). These results suggest that Cs-ME displays skin protection effects via the control of EKR/p38-AP-1 signaling.

## 4. Discussion

In this study, we examined the photoprotective and skin barrier-strengthening effects of Cs-ME. To investigate the photoprotective ability, the influence of Cs-ME on cell death and skin aging-related gene expression as well as ROS generation was assessed in 30 mJ/cm^2^ UVB-irradiated HaCaT cells. To study the role of Cs-ME on skin barrier function, mRNA expression of the skin barrier components FLG, TGM-1, and HAS was measured in Cs-ME-treated HaCaT cells. The effect of Cs-ME on AP-1 signaling was also explored.

UV exposure is known to influence skin physiology by cell death due to cell damage [34]. In this study, living cell counting assays and cell viability assays revealed that Cs-ME blocked cell death invoked by UV irradiation (Figure 1(a)). When the skin is exposed to UV, ROS including H_2_O_2_ and OH radicals increase within 15 min and can last up to 60 min [35]. A moderate amount of ROS acts as a second messenger and regulates various signal transduction pathways to perform important functions in various physiological responses, such as cell proliferation, dermal angiogenesis, wound healing, and skin repair [36, 37]. However, UV-induced aberrant ROS leads to oxidative stress and DNA damage [38–40]. Based on these reports and the cell viability results experiments shown in Figure 1, we predicted that the effect of Cs-ME, which protects cells from UV-induced cell death, would be dependent on free radical inhibition. The antioxidant effect of Cs-ME was evaluated using DPPH and ATBS assays, which are performed in cell-free systems. The ROS generating assays in HaCaT cells showed that Cs-ME has radical scavenging activity (Figures 2(a), 2(b), and 2(c)). In addition, our prediction was validated by observing that Cs-ME reduced cell death, even under conditions in which H_2_O_2_ directly increased ROS generation (Figure 2(d)).

Since ROS can enhance the expression of enzymes that degrade the extracellular matrix (ECM) of the dermis to form wrinkles and cause the skin to age [14], the effects of Cs-ME on skin aging have also been studied. It was reported that UV exposure and generation of excessive ROS can contribute to skin aging by triggering the activation of HYALs and MMPs [32, 41]. However, since the expression pattern of HYALs and MMPs is different depending on the type of cell [42–45], we first identified which HAYL and MMP proteins were increased under our experimental conditions. As a result of PCR analysis, only increased expression of HYAL-1 and MMP-1 was observed among all the HYAL and MMP genes in UV-irradiated HaCaT cells (Figure 3(a)). Interestingly, the Cs-ME treatment suppressed HYAL-1 and MMP-1 gene expression in HaCaT cells that were stimulated by UV or H_2_O_2_ (Figure 3(c)), suggesting that Cs-ME exerts antiaging ability through ROS regulation. Antioxidants have been widely used to reverse skin aging. For example, idebenone, a synthetic analog of coenzyme Q 10 with strong antioxidant ability, has been used as a component of cosmetics for the improvement of photoaging skin [46]. Another representative antioxidant, vitamin C, also improved clinically photo-aged skin [47]. Thus, Cs-ME has the potential for use as an ingredient for antiaging cosmetics based on its antioxidant effects.

Our study also highlights the role of Cs-ME in the regulation of skin barrier molecules, such as FLG and HAS proteins. FLG, an essential structural protein of the epidermis, is known to play an important role in maintaining skin moisture [48]. Deaminated FLG is degraded to release hygroscopic amino acids, such as arginine and histidine, and a mixture of these hygroscopic amino acids is involved in moisturizing the skin by forming a “natural moisturizing factor” [49]. HAS molecules are known for retaining skin moisture [50]. Interestingly, Cs-ME specifically increased the mRNA expression of FLG and HAS-1 (Figure 4(a)). In addition, AP-1 luciferase activity and phosphorylation of ERK and p38 were enhanced by Cs-ME. A previous study demonstrated that the expression of FLG is dependent on ERK-AP-1 signaling in normal human epidermal keratinocytes (NHEKs) [51]. The p38 MAPK pathway has been shown to be important for the induction of HAS-1 expression in human fibroblast-like synoviocytes [52, 53]. Based on these previous reports, we predicted that Cs-ME would increase FLG and HAS-1 expression through ERK-AP-1 and p38-AP1 signaling activation, respectively. Furthermore, HAS molecules have been reported to be unbalanced in atopic dermatitis (AD). The expression of HAS-1 was shown to be decreased and HAS-3 increased, while HAS-2 expression was almost unchanged in AD skin lesions suggesting that distinct HASs are differentially regulated and that HAS-1 plays a major role in HA synthesis in the AD pathological condition [54]. FLG was also reduced in the skin and keratinocytes of patients with ichthyosis vulgaris or AD [55–57]. Thus, future studies are required to elucidate the therapeutic effect of Cs-ME on skin diseases. In conclusion, Cs-ME, which promotes FLG and HAS-1 expression, not only strengthens the skin barrier and skin hydration but also is expected to relieve symptoms in pathological conditions such as AD.

**Figure 5:**
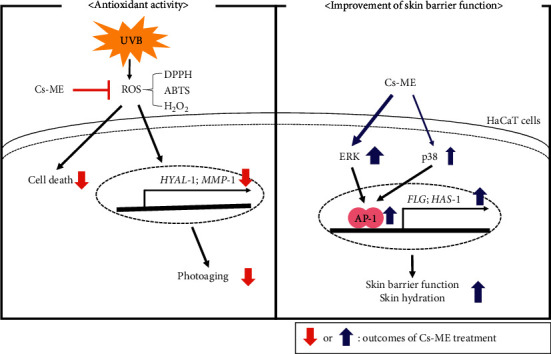
Schematic diagram of the antioxidant and skin protective effects of Cs-ME.

Taken together, we demonstrated that Cs-ME has cytoprotective activity and antiaging capacity via ROS inhibition in UV irradiation conditions. Furthermore, Cs-ME exerts the ability to protect the skin barrier and enhance skin hydration by elevating FLG and HAS-1 expression through modulation of ERK-AP-1 signaling and p38-AP-1 signaling, as summarized in Figure 5. Thus, Cs-ME is anticipated to be an effective ingredient for cosmetics to prevent skin aging, maintain moisture, and improve the skin barrier. A lot of skin barrier-related functions such as permeability, antimicrobial activity, psychosensory and neurosensory interfaces, cohesion (integrity), and mechanical or rheological protection are not yet tested with Cs-ME. Therefore, further tests for those functions will proceed in the following studies.

## Figures and Tables

**Table 1:** Sequence of PCR primers used in this study.

Target	Sequence (5' to 3')
*HYAL-*1	
Forward	CAGAATGCCAGCCTGATTGC
Reverse	CCGGTGTAGTTGGGGCTTAG

*HYAL-*2	
Forward	TACACCACAAGCACGGAGAC
Reverse	ATGCAGGAAGGTACTGGCAC

*HYAL-*3	
Forward	CCAGGATGACCTTGTGCAGT
Reverse	CCATCTGTCCTGGATCTCGC

*MMP-*1	
Forward	TCTGACGTTGATCCCAGAGAGCAG
Reverse	CAGGGTGACACCAGTGACTGCAC

*MMP-*3	
Forward	ATCCTACTGTTGCTGTGCGT
Reverse	CATCACCTCCAGAGTGTCGG

*MMP-*9	
Forward	GCCACTTGTCGGCGATAAGG
Reverse	CACTGTCCACCCCTCAGAGC

*FLG*	
Forward	AGGGAAGATCCAAGAGCCCA
Reverse	ACTCTGGATCCCCTACGCTT

*TGM-*1	
Forward	GAAATGCGGCAGATGACGAC
Reverse	AACTCCCCAGCGTCTGATTG

*HAS-*1	
Forward	CCACCCAGTACAGCGTCAAC
Reverse	CATGGTGCTTCTGTCGCTCT

*HAS-*2	
Forward	TTCTTTATGTGACTCATCTGTCTCACCGG
Reverse	ATTGTTGGCTACCAGTTTATCCAAACG

*HAS-*3	
Forward	TATACCGCGCGCTCCAA
Reverse	GCCACTCCCGGAAGTAAGACT

*GAPDH*	
Forward	GGTCACCAGGGCTGCTTTTA
Reverse	GATGGCATGGACTGTGGTCA

## Data Availability

The data used to support the findings of this study are available from the corresponding author upon request.

## Abbreviations

UV: Ultraviolet
ROS: Reactive oxygen species
NOS: Nitric oxide synthase
PKC: Protein kinase C
FLG: Filaggrin
HA: Hyaluronic acid
HAS: Hyaluronic acid synthase
AP-1: Activator protein 1
DPPH: 1,1-Diphenyl-picrylhydrazyl
ABTS: 2,2′-Azino-bis(3-ethylbenzothiazoline-6-sulfonic acid)
TGM-1: Transglutaminase-1
ERK1/2: Extracellular signal-regulated kinases 1 and 2
JNK: c-Jun N-terminal kinase
AP-1: Activation of activator protein-1
ECM: Extracellular matrix
NMSC: Nonmelanoma skin cancer
BCC: Basal cell carcinoma
AD: Atopic dermatitis.
